# Increased Absolute Glutamate Concentrations and Glutamate-to-Creatine Ratios in Patients With Methamphetamine Use Disorders

**DOI:** 10.3389/fpsyt.2018.00368

**Published:** 2018-08-31

**Authors:** Wenhan Yang, Ru Yang, Jing Luo, Lei He, Jun Liu, Jun Zhang

**Affiliations:** ^1^Department of Radiology, Second Xiangya Hospital of Central South University, Changsha, China; ^2^Hunan Judicial Police Vocational College, Changsha, China

**Keywords:** glutamate, magnetic resonance spectroscopy, substance use disorders, neural circuits, neurotransmitters, methamphetamine, addiction

## Abstract

**Introduction:** Previous studies have indicated that changes in the concentration of glutamate and related metabolites may mediate the progression of addiction in patients with methamphetamine (MA) use disorders. In the present study, we utilized magnetic resonance spectroscopy (MRS) to investigate absolute glutamate concentrations and metabolite ratios in patients with MA addiction. We further analyzed the association between glutamate concentration and various clinical indicators.

**Methods:** The present study included 31 unmedicated patients with clinically diagnosed MA dependence (mean age: 30.5 ± 8.0 years) and 32 age-matched healthy controls (mean age: 32.9 ± 8.2 years). Patients were evaluated using the Barratt Impulsiveness Scale (BIS-11). We also collected general information regarding the duration and dosage of drug use. Point-resolved spectroscopy was used to quantify the absolute concentrations of metabolites (glutamate, choline, N-acetylaspartate, glutamine, and creatine), as well as the ratio of metabolites to total creatine, using LCModel software. We then compared differences in glutamate levels and psychometric scores between the two groups.

**Results:** Glutamate-to-creatine ratios in the brainstem were significantly higher in the MA group than in the control group (*t* = 2.764, *p* = 0.008). Glutamate concentrations in the brainstem were also significantly higher in the MA group than in the control group (*t* = 2.390, *p* = 0.020). However, no significant differences in the concentrations or ratios of other metabolites were observed between the two groups (all *p* > 0.05). Glutamate concentration was positively correlated with the duration of drug use (*r* = 0.401, *p* = 0.035) and the total dose of regular addiction (duration of addiction × regular addiction dose; *r* = 0.207, *p* = .040), but not with BIS-11 scores.

**Conclusions:** Our findings indicated that glutamate levels in the brainstem are significantly elevated in patients with MA use disorders, and that these levels are significantly associated with the duration and dose of drug use.Such findings suggest that glutamate concentration can be used as an objective biological marker for evaluating/monitoring disease status and treatment efficacy in patients with MA dependence.

## Introduction

Methamphetamine (MA) is a highly addictive, widely abused psychostimulant with severe neurotoxic potential (1). Although MA was discovered decades ago, it has recently become one of the most widely used drugs in the world. MA abuse can lead to the emergence and spread of a variety of diseases, increasing the risk of HIV, hepatitis B, hepatitis C, and other diseases due to the sharing of syringes. Additional studies have demonstrated that long-term MA use can lead to elevated blood pressure due to excitation of the sympathetic nervous system, as well as impairments in brain structure, function, and cognition (2). Thus, MA abuse and dependence represent serious public health concerns. However, the diagnosis of MA use disorders has primarily been based on descriptive, symptomatic checklist criteria, and the neural mechanisms underlying the highly addictive nature of the drug remain to be fully elucidated. Further research is required to identify biological markers of MA addiction, and to develop more effective means of monitoring disease status and evaluating the efficacy of therapeutic interventions in patients with MA dependence.

Amphetamine-like psychostimulants are characterized by their ability to bind to dopamine transporters (DATs) (3), which transfer monoamines released into the synapse back into the cytosol, including dopamine. MA binds more rapidly to DAT and is thus more toxic than other amphetamine-like psychostimulants. Although the dopaminergic system is closely related to the formation of addiction, amphetamines are also associated with the adaptation of glutamate signaling (4). Upon entering dopaminergic neurons, amphetamines stimulate endocytosis of the excitatory glutamate transporter EAAT3.

As the main excitatory neurotransmitter in the human central nervous system (CNS), glutamate is involved in a variety of physiological processes. Previous studies have demonstrated that excessive endogenous glutamate is associated with several acute and chronic neurodegenerative diseases (5). Several previous studies have reported that patients with MA addiction exhibit altered levels of glutamate metabolites in the brain. Because glutamate is a key element of the brain's reward system, it may play an intermediate role in the process of addiction. Treatment with N-acetylcysteine (NAC) normalizes glutamate homeostasis and prevents relapse in drug-dependent animals (6). However, the effects of substance addiction on glutamate metabolism in human patients remain unknown. Proton magnetic resonance spectroscopy (1H-MRS) findings regarding brain levels of glutamate in individuals with substance use disorders have been mixed. Some studies have reported that glutamate levels are decreased in patients with alcohol addiction, while others have reported conflicting results. Mon et al. reported that individuals with alcohol dependence exhibited lower baseline concentrations of glutamate in the anterior cingulate cortex than those in the control group (7). Similarly, Bagga et al. reported that glutamate levels were significantly lower in the primary visual cortex in patients with alcohol dependence than in healthy controls (8). However, Frye et al. observed that patients with alcohol dependence exhibited significantly elevated levels of glutamate in the midline anterior cingulate cortex when compared with controls (9). Hermann D et al. found significantly increased glutamate levels during acute alcohol withdrawal in corresponding prefrontal cortex regions of treatment-seeking alcoholic patients and alcohol-dependent rats compared with respective control subjects (10).

When investigating traditional drug dependence, Schmaal et al. observed significant increases in glutamate levels in the anterior cingulate cortex of cocaine users, relative to those observed in healthy controls. Using high-speed amperometry with enzyme-based biosensors, Wakabayashi, and Kiyatkin discovered that an initial intravenous injection of cocaine induces rapid, transient increases in glutamate release in the nucleus accumbens (NAc) of freely moving rats. Moreover, subsequent injections rapidly strengthened this response (11). However, few studies have examined changes in glutamate levels due to the use of synthetic drugs.

White et al. (12) examined the concentration of glutamine metabolites in 26 healthy individuals following oral administration of single, clinically relevant doses of amphetamine (20 mg), MA (20 mg), or placebo. Using MRS, the authors revealed that d-amphetamine administration increased levels of glutamate, glutamine, and creatine in the dorsal anterior cingulate cortex (dACC) 3 days after the peak drug reaction (140–150 min post-ingestion).

In the present study, we aimed to investigate the absolute concentration and ratio of glutamate to other metabolites in patients with MA addiction, in order to aid in the development of a biological marker for evaluating/monitoring disease status as well as the efficacy of therapeutic interventions.

## Materials and methods

### Participant characteristics

The present study included 31 unmedicated individuals with MA addiction from Changsha (Hunan Province) undergoing treatment at a drug rehabilitation center in Zhuzhou. All participants provided written informed consent. The experiment was approved by the ethics committee of the Second Xiangya hospital of Central South University. Inclusion criteria were as follows: (1) positive urine test for MA; (2) diagnosis of addiction based on criteria outlined in the fourth edition of the Diagnostic and Statistical Manual of Mental Disorders (DSM-IV), (3) negative urine test for heroin and ketamine; (4) no history of structural brain disease, epilepsy, or head trauma; (5) no contraindications for MRI; (6) no history of mental or psychiatric illness. Patients with a history of serious physical illness, intracranial lesions, mental illness, or MRI contraindications were excluded. Patients meeting the aforementioned criteria underwent MRI examination after 5–30 days of abstinence. We recruited 33 control participants through WeChat, flyers, etc. All participants were right-handed and of Han Chinese descent. Most participants were smokers, while some reported alcohol use as well. Barratt Impulsiveness Scale (BIS-11) scores were obtained for all patients (13) following MRI examination. The demographic characteristics of the experimental and control groups are shown in Table 1.

**Table 1:** Participant characteristics.

	**Patients (*n* = 31) (mean ±*SD*)**	**Controls (*n* = 32)(mean ±*SD*)**	***χt*/χ^2^ /Z value**	***p***
Age (years)	30.5 ± 8.0	32.9 ± 8.2	*t* = -1.139	0.259
**GENDER (*****N*****)**				
Male	21	20	χ^2^ = 0.190	0.793
Female	10	12		
**EDUCATION (*****N*****)**				
Primary school	3	2	Z = -0.377	0.706
Junior high school	20	21		
Senior high school	8	9		
**NICOTINE USE (*****N)***				
Y	29	26	χ^2^ = 3.506	0.257
N	2	6		
FTND	6.1 ± 1.6	5.7 ± 1.5	*t* = -0.78	0.439
**ALCOHOL USE (N)**				
Y	12	7	χ^2^ = 2.119	0.177
N	19	25		
AUDI	7.6 ± 1.8	6.9 ± 3.4	*t* = 0.580	0.615

*FTND, Fagerstrom Test for Nicotine Dependence; AUDI, Alcohol Use Disorders Identification Test*.

### Acquisition/analysis of MRS data

**Figure 1:**
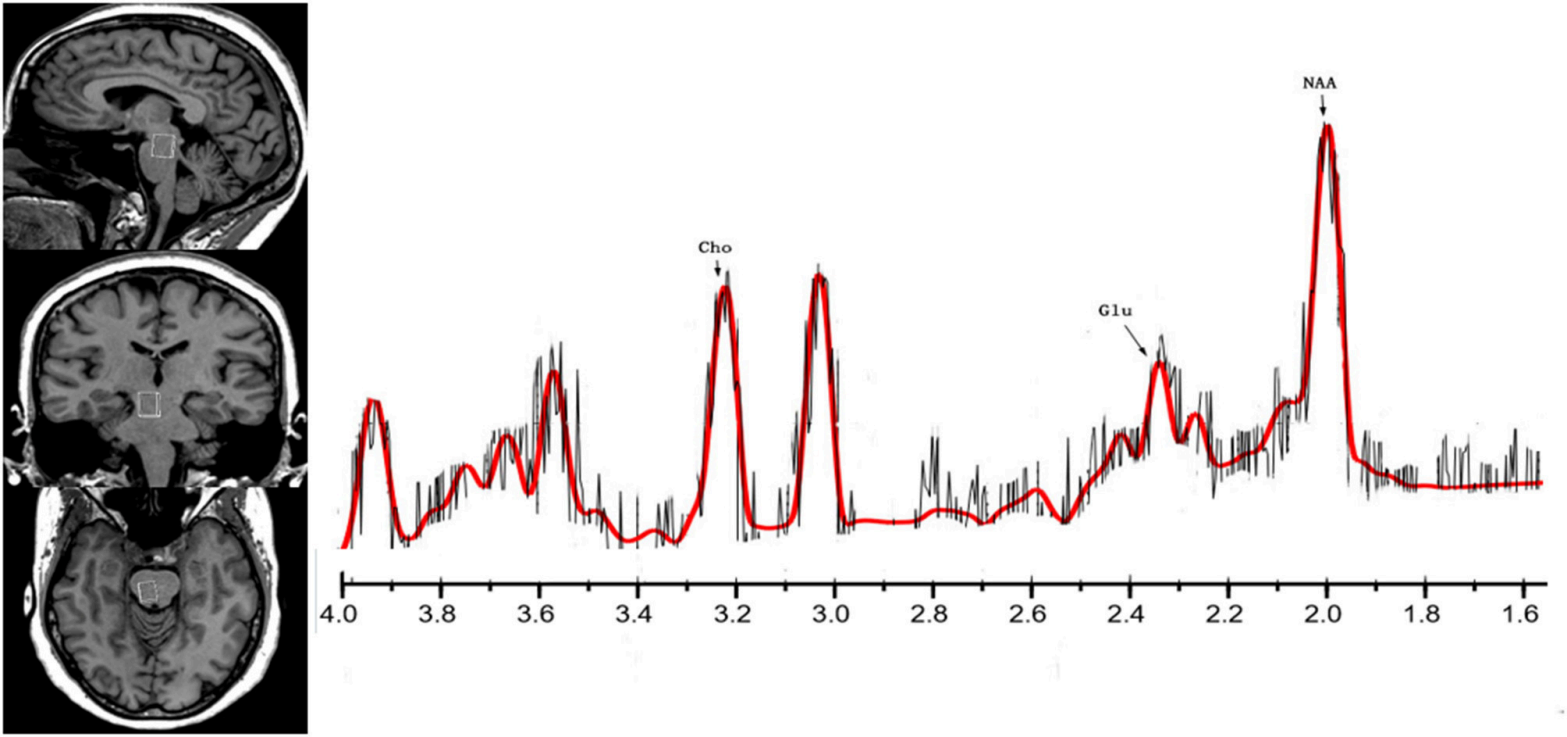
The location and MR spectra of the region of interest in the brainstem on the left side, the localized images of the brainstem from top to bottom are, in order, sagittal, coronal, axial, the interest area located in the midbrain aqueduct in the coronal plane. The result of MR spectra of the right brainstem in the patient (red line) brain is on the other side.

All participants underwent imaging in a 3T MRI scanner (MAGNETOM Skyra, Siemens) equipped with a 32-channel phased-array joint head coil. Foam padding and a forehead- restraining strap were utilized to limit head movement during the scanning procedure. To obtain high-quality spectroscopy data, participants were advised regarding the importance of remaining motionless during the procedure. All participants were allowed a moment to relax and move their hands/feet prior to scanning to ensure the quality of MR images. High-resolution, T1-weighted anatomical images of the whole brain were acquired using a three-dimensional fast gradient echo sequence with the following parameters: repetition time (TR) = 1,450 ms, echo time (TE) = 2.0 ms, inversion time (TI) = 900 ms, field-of-view (FOV) = 256 × 256 mm, slice thickness = 1 mm, flip angle = 12°. Slices in the three orthogonal planes were subjected to multiplanar reconstruction in order to localize the volumes of interest (VOI: 10 × 12 × 15 mm = 1.8 cm3). The VOI was positioned to cover the right brainstem in coronal and sagittal slices as a topographic marker (Figure 1).

All MRS data were acquired using single-volume localization. Spectral data were acquired via conventional point-resolved spectroscopy (PRESS) using a short TE to ensure optimal selectivity for glutamate. These spectra were also collected with a TR of 1290 ms (average: 240), resulting in a total scan time of over 6.75 min with and without water suppression. Raw data from each acquisition, which consisted of 1,024 points, were collected at a bandwidth of 1,200 Hz. The total examination lasted approximately 10 min for each participant. Spectra were quantified using LCModel software(14, 15).

Cramer–Rao lower bounds (CRLBs) were used to evaluate the accuracy of the amplitude calculation for each component. CRLB estimates represent the %SD of the fit for each metabolite. Only metabolite concentrations with CRLBs below 20% (indicate of high confidence) were accepted and used for the following analyses.

### Statistical analysis

Statistical analyses were performed using SPSS 18.0. Demographic characteristics were compared using independent Samples *t*-tests, chi-square tests, and rank sum tests. Differences in metabolite levels were analyzed using two-sided, independent Samples *t*-tests and rank sum tests. The data distributions were tested for normality using the Kolmogorov–Smirnov test. Normally distributed data were analyzed using independent Samples *t*-tests, while non-normally distributed data were analyzed using rank sum tests. Correlations between metabolite levels and clinical characteristics were evaluated using Pearson's correlation coefficients. The level of statistical significance was set at *p* < 0.05.

## Results

### MRS relative metabolite concentrations

**Figure 2:**
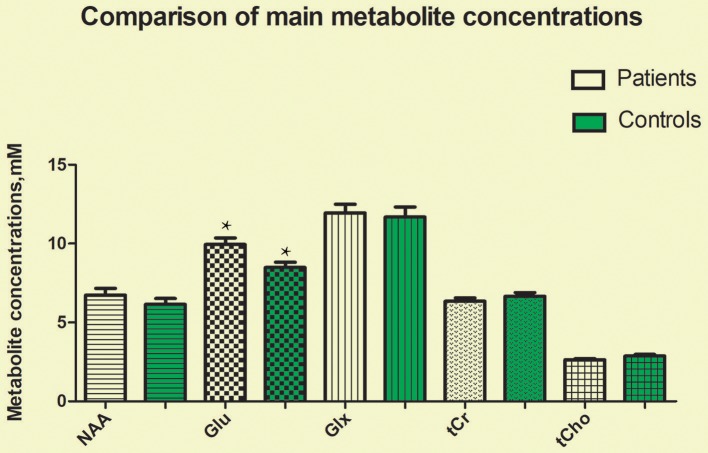
Glutamate concentrations in the brainstem were significantly higher in the MA group than in the control group (*t* = 2.390, *p* = 0.020). No significant differences in the concentrations of other metabolites were observed between the two groups (all *p* > 0.05). ^*^means the existence of statistical differences between the two groups.

**Figure 3:**
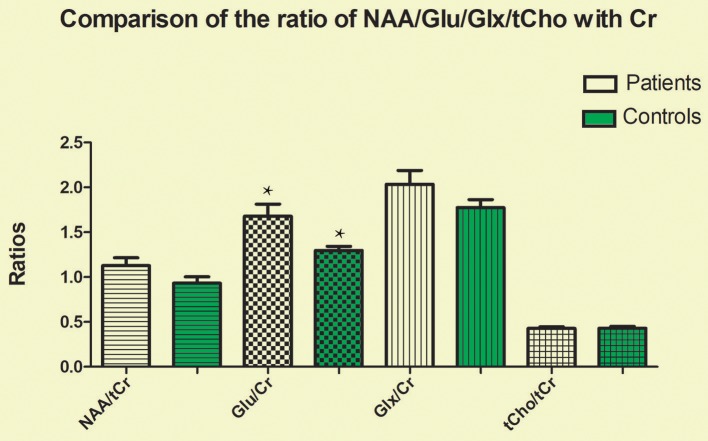
Glutamate-to-creatine ratios in the brainstem were significantly higher in the MA group than in the control group (*t* = 2.764, *p* = 0.008). No significant differences in the ratios of other metabolites were observed between the two groups (all *p* > 0.05). ^*^means the existence of statistical differences between the two groups.

The CRLBs of N-acetylaspartate (NAA), glutamate, creatine, and choline were less than 20% for all participants. Glutamate, **glx (glutamate + glutamine)**, and choline data were excluded for two, two, and three participant of the MA group due to excessive CRLBs. **Glutamine data were not analyzed for the excessive CRLBs of some part of data**. The mean metabolite concentrations in the brainstem are displayed in Table 2, Figures 2, Figures 3. Our findings indicate that glutamate concentrations were higher in the MA group than in the control group [*t*_(57)_ = 2.390, *p* = 0.020]. The ratio of glutamate to creatine was also significantly higher in the MA group than in the control group [*t*_(57)_ = 2.764, *p* = 0.008]. Although the mean **glx (glutamate + glutamine)** concentration was higher in the MA group than in the control group, this difference was not significant. No significant differences in levels of other metabolites were observed between the two groups (all *p* > 0.05).

**Table 2:** Comparison of major metabolites in the brainstem.

**Metabolite**	**Patients (mean ± *SD*)**	**Controls (mean ± *SD*)**	***t***	***p***
NAA	6.25 ± 2.23	6.14 ± 2.13	*t*_(59)_ = -0.188	0.851
Glu	9.94 ± 2.16	8.48 ± 1.89	*t*_(59)_ = 2.390	0.020
Glx	11.94 ± 2.73	11.69 ± 3.61	*t*_(59)_ = -2.98	0.767
tCr	6.56 ± 1.07	6.66 ± 1.37	*t*_(61)_ = 0.292	0.772
tCho	2.64 ± 0.33	2.87 ± 0.61	*t*_(58)_ = 1.657	0.103
NAA/tCr	1.13 ± 0.37	1.13 ± 0.48	*t*_(59)_ = -0.188	0.851
Glu/tCr	1.68 ± 0.73	1.30 ± 0.27	*t*_(59)_ = 2.764	0.008
Glx/tCr	2.03 ± 0.83	1.77 ± 0.50	*t*_(59)_ = -0.149	0.143
tCho/tCr	0.43 ± 0.12	0.43 ± 0.16	*t*_(58)_ = 0.115	0.909

*NAA, N-acetyl aspartate; Glu, glutamate; Glx, glutamate+glutamine; tCr, total creatine; tCho, total choline*.

*Patients: NAA n = 31, Glu n = 29, Glx n = 29, tCr n = 31, tCho n = 29*.

*Controls: NAA n = 32, Glu n = 32, Glx n = 32, tCr n = 32, tCho n = 31*.

### Duration of MA use

**Figure 4:**
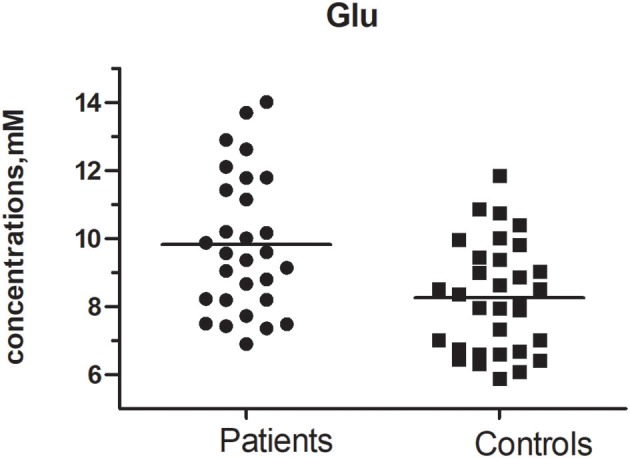
Glu concentration between MA and Control group (*t* = 2.390, *p* = 0.020).

**Figure 5:**
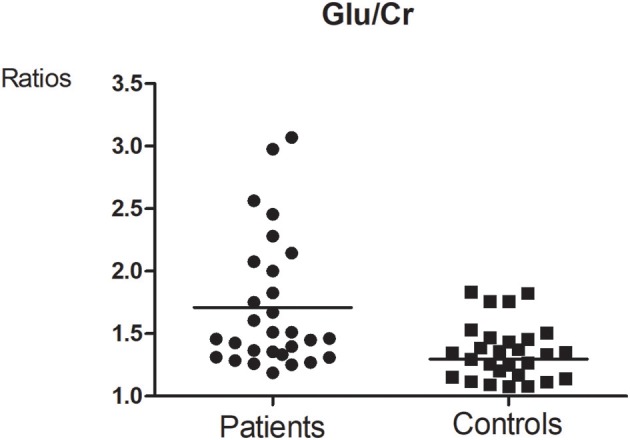
Glutamate-to-creatine ratios between MA and Control group (*t* = 2.764, *p* = 0.008).

**Figure 6:**
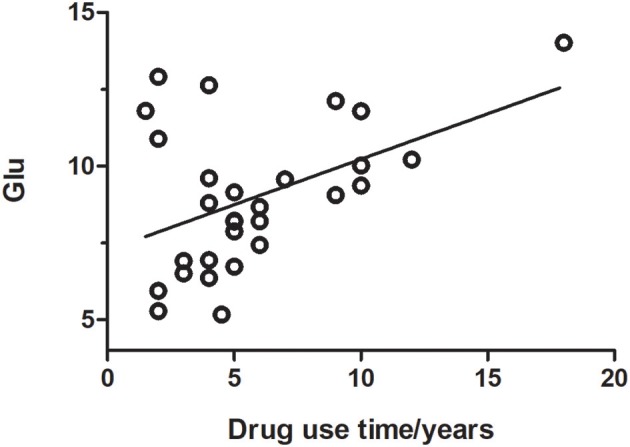
Glutamate concentration was positively correlated with the duration of drug use (*r* = 0.401, *p* = 0.035).

Glutamate concentrations and glutamate-to-creatine ratios were significantly higher in patients than controls (Figures 4, 5). In addition, we observed a significant positive correlation between absolute concentrations of glutamate and the duration of MA use (Figure 6; *r* = 0.401, *p* = 0.035). Glutamate concentration increased with years of MA use and was also associated with the total dose of regular addiction (duration of addiction × regular addiction dose; *r* = 0.207, *p* = 0.040). Slight negative correlations were also observed between glutamate levels and BIS-11 scores (*r* = −0.148, *p* = 0.291). No significant correlations were observed between levels of other metabolites and clinical characteristics.

## Discussion

In the present study, we compared absolute glutamate concentrations and metabolite ratios between patients with MA addiction and healthy controls. Our findings indicated that glutamate concentrations were significantly higher in patients than controls, and that absolute glutamate concentrations were correlated with the duration of MA use.

Glutamate is a vital excitatory neurotransmitter in the brain. The excitatory toxicity of glutamate is related to the pathogenesis of various neurological diseases, such as stroke, amyotrophic lateral sclerosis, and epilepsy (16). Several recent studies have suggested that glutamate is associated with synaptic plasticity in patients with substance addiction (17–20). In addition to its direct influence as an excitatory amino acid, glutamate plays an important role in long-term neuronal enhancement, and is closely related to learning and memory (21, 22). Plasticity of glutamate synapses may be closely related to the long-term process of addiction. Addiction results in long-term alterations to the structure and function of the brain (23, 24), and previous research has indicated that changes in glutamate-mediated neuroplasticity in the reward pathway are critical in the formation of addictive memories, which in turn increase the likelihood of relapse (25). Addictive drugs influence learning and memory by directly altering dopamine neurotransmission in the midbrain, which leads to subsequent changes in the dorsal and ventral striatum, resulting in increased addiction memory consolidation (26).

The brainstem includes the midbrain, pons, and medulla. The midbrain is an important part of the brain's reward system. The ventral tegmental area (VTA) plays a central role in motivation and reward (27). Addictive drugs are taken up by neurons in the VTA, resulting in adaptation of glutamatergic synapses as well as changes in the dopamine system of the midbrain (28). In addition, the pedunculopontine tegmental nucleus (PPTg) of the brainstem contains neurons that release glutamate, acetylcholine, and GABA, thereby influencing activity in the basal ganglia and limbic region (29–31). Previous studies have indicated that glutamate neurons in the ppTg are directly controlled by the VTA, which has been associated with neural regulation in the reward circuit (32). In accordance with previous findings, our results indicated the glutamate levels in the brainstem were higher in the MA group than in the control group. Such findings suggest that elevated glutamate concentrations and glutamate-to-creatine ratios in the brains of patients with MA addiction are associated with changes in synaptic plasticity and the retention of addictive memories. Indeed, it is now widely accepted that increases in extracellular glutamate levels mediate MA-induced neurotoxicity (33). Our results support the hypothesis that glutamate mediates the neuro-modulatory system and reward circuits involved in addiction.

Changes in the neuro-modulatory systems and neural circuits for reward can lead to distinct psychiatric disorders, including addiction (34). Drug addiction is associated with increased dopamine concentrations in the synaptic cleft (35). Neuronal changes in the limbic system can alter behavioral responses to various environmental stimuli associated with reward behavior. Psychostimulants and drug abuse can cause significant synaptic changes in the middle cerebral dopamine system (36). Dopamine is transferred from the ventral tegmental area (VTA) in the midbrain—a process associated with motivation and drug addiction (37).

Drug addiction is a severe mental illness characterized by compulsive drug abuse despite the potential undesirable consequences. Previous studies have indicated that the use of psychoactive stimulants may lead to changes in a wide range of neurological circuits and physiological processes (38). Psychostimulants play a significant role in promoting the structural plasticity of reward circuits in the brain (39). Addiction interacts with the reward system to motivate drug-seeking behavior, inhibit self-control mechanisms, and promote compulsive drug use (40). Such findings may explain the high rate of relapse in patients with drug addiction.

So far, what is known to us is that long-term MA use can lead to structural and functional changes in the brain. The brain's glutamate system plays a key role in long-term plasticity associated with learning and memory. Patients with MA use disorders exhibit persistent cognitive impairments and neurological deficits, which may be related to changes in the prefrontal cortex (PFC) and its glutamatergic projections to the NAc (41). Most previous studies have indicated that the progression of drug addiction is largely due to the adaptive neurobiological response to drug abuse in the corticostriatal glutamatergic and dopaminergic systems in the brain (42, 43). Thus, repeated drug abuse may lead to neurobiological adaptations that promote habitual drug use (44).

Some previous MRS studies have reported that patients with MA use disorders exhibit decreases in NAA/creatine levels, as well as increases in levels of inositol (45, 46). However, in the present study, we observed no significant differences in the concentrations of these metabolites between the MA and control groups. But there are still deficiencies in our experiment. The parameter TE = 33 ms was set, which is a little bit different from the basis data provided for LCModel TE = 30 ms and that is a limitation. To display glx complex (glutamate + glutamine) better, preliminary experiment was designed to detect the optimum echo time (TE). Because of the default setting of TE in LCModel software is 30 ms, 4 TEs around 30 ms (27,30,33, and 36ms) were set. The result shows that the CRLBs (Cramer-Rao) is the lowest and the SNR is the highest when TE = 33 ms. The preliminary experiment also has a limitation that there were only a few subjects in each group. Further studies are required to verify our findings. Another limitation is that there were six matched subjects who could not remember their drug use time accurately, so other six subjects before matched were replaced in Figure 6. In addition, changes in the concentrations of glutamate have been implicated in the neuroadaptation processes associated with drug addiction (47). However, such measurements are difficult to obtain due to the overlapping resonance of glutamate and glutamine. We used LCModel software to separate glutamate signals. Our findings indicated that glutamate concentration was higher in the MA group than in the control group, consistent with the findings of Sailasuta et al. (48).

Nonetheless, findings regarding metabolite concentrations in the brains of MA users remain inconsistent. In a 1H-MRS study of 29 patients with MA addiction and 45 healthy controls, Crocker et al. discovered that patients of the MA group exhibited decreased levels of glutamate (49, 50). In contrast to our findings, one previous study involving 44 adolescent MA users and 53 healthy adolescents reported that NAA levels were lower in the anterior cingulate cortex of teenagers in the MA group. Such differences may be due to differences in participant characteristics. Naaijen et al. (51) discovered that glutamate + glutamine (glx) signals were decreased in the anterior cingulate cortex of pediatric patients with autism spectrum disorders (ASD) and attention deficit hyperactivity disorder (ADHD). However, Glx signals were lower in adults with ASD and ADHD, indicating that neurodevelopmental changes in prefrontal glutamate concentration occur throughout the life cycle. Ernst and Chang reported that short-term abstinence from MA use is associated with dynamic abnormalities in glx levels (52), suggesting that normalization of Glx levels may reduce cravings for MA.

### Conclusion

Our findings indicated that glutamate concentrations and glutamate-to-creatine ratios were significantly higher in patients with MA use disorders than in controls, and that absolute glutamate concentrations were correlated with the duration of MA use. Such findings may aid in the development of objective biological markers for evaluating/monitor disease status and treatment efficacy in patients with MA dependence.

## Data availability statement

The raw data supporting the conclusions of this manuscript will be made available by the authors, without undue reservation, to any qualified researcher.

## Author contributions

WY and RY designed the study. JiL and LH conducted the assessments. JZ conducted behavioral and imaging analyses. JuL modified the manuscript and supervised the study. WY wrote the first draft and all authors provided input to the final version of the manuscript.

### Conflict of interest statement

The authors declare that the research was conducted in the absence of any commercial or financial relationships that could be construed as a potential conflict of interest.
